# The future of STI screening and treatment for youth: a National Survey of youth perspectives and intentions

**DOI:** 10.1186/s12889-021-12091-y

**Published:** 2021-11-04

**Authors:** Vinaya Gogineni, Marika E. Waselewski, Cornelius D. Jamison, Jasmine A. Bell, Nicole Hadler, Kiren A. Chaudhry, Tammy Chang, Okeoma O. Mmeje

**Affiliations:** 1 The University of Toledo College of Medicine and Life Sciences, 3000 Arlington Ave, Toledo, OH 43614 USA; 2 University of Michigan Department of Family Medicine, 1500 E. Medical Center Dr, Ann Arbor, MI 48109 USA; 3 University of Michigan Institute for Healthcare Policy and Innovation, 2800 Plymouth Rd. Bldg. 16, Ann Arbor, MI 48109 USA; 4 University of Michigan Department of Obstetrics and Gynecology, 1500 E. Medical Center Dr., L4100 Women’s Hospital, Ann Arbor, MI 48109 USA; 5 University of Michigan Medical School, 1301 Catherine St, Ann Arbor, MI 48109 USA; 6 Department of Health Behavior and Health Education, University of Michigan School of Public Health, 415 Washington Heights, Ann Arbor, MI 48109 USA; 7 National Clinician Scholars Program, University of Michigan, 2800 Plymouth Rd. Bldg. 16, Ann Arbor, MI 48109 USA

**Keywords:** Sexual and reproductive health, Text message, School-based health centers, Adolescent health

## Abstract

**Background:**

Sexually transmitted infection (STI) rates continue to rise in the U.S., with disproportionately high rates among those aged 15–24 years. Effective programs and policies are necessary to address this growing public health problem. The purpose of this study is to assess the perspectives of a national sample of youth on access to STI care and behaviors regarding STIs.

**Methods:**

MyVoice, a national text message survey of youth, was used to pose four open-ended questions on STI screening and treatment to 1115 youth aged 14–24 in August 2018. A mixed-methods strategy was employed for the study. Qualitative data was analyzed using a modified grounded theory approach. Summary statistics were calculated for demographic data and prevalence of themes.

**Results:**

Of the 800 participants who responded to at least one question (72% response rate), mean age was 19 years (SD = 3.1), 55% identified as female, 61% identified as non-Hispanic white, and 33% qualified for free/reduced lunch. A majority felt it would be easy to get screened (69%) or treated (68%) for an STI. Nearly all respondents (95%) stated they would share an STI diagnosis with their sexual partners.

**Conclusions:**

Despite high rates of STIs among youth, most respondents reported that STI screening and treatment is accessible, and they would share an STI diagnosis with their partner.

**Supplementary Information:**

The online version contains supplementary material available at 10.1186/s12889-021-12091-y.

## Background

The continuous rise in sexually transmitted infections (STIs), specifically among youth, raises concerns for the U.S. population’s reproductive health outcomes. The Centers for Disease Control and Prevention’s (CDC) annual STI surveillance report in 2018 revealed more than 1.7 million cases of *Chlamydia trachomatis* (CT) and more than 583,000 cases of *Neisseria gonorrhoeae* (NG) in the United States [1]. Youth aged 15–24 years account for nearly half of the new cases of STIs due to riskier sexual behavior such as multiple concurrent partners or unprotected intercourse and lower access to sexual healthcare [2]. Recurrent or untreated CT and NG infections increase the risk of pelvic inflammatory disease in women and infertility and HIV infection in men and women. Additionally, there are increasing rates of antibiotic resistance in NG infections, which affect both men and women [1]. Therefore, it is critical that effective preventive measures be utilized to prevent STI transmission and STI-related morbidity.

Understanding youth perceptions and behaviors regarding STIs is a crucial first step in the development and implementation of effective STI treatment and prevention methods for this population [2]. Despite the high incidence of CT and NG, a 2016 national survey of youth aged 15–25 years reported only 16.6% of female respondents and 6.6% of male respondents had received STI screening within the past year [2]. Previous work has suggested potential barriers to youth STI screening and according to a 2013–2015 national survey, youth aged 15–25 years had concerns about privacy and confidentiality that hindered many individuals from obtaining STI screening [3]. In some cases, youth do not believe they are at risk for contracting an STI and therefore do not seek these sexual health services [4]. However, there is currently a lack of research that addresses youth-specific STI interventions [2]. This gap in literature introduces an opportunity for our study to provide new insight on youth perceptions of STI testing and treatment, access to care, and notification of partners.

We sought to understand youth perceptions of their access to STI screening and treatment to better shape the delivery of reproductive healthcare services to youth populations. We posed open-ended questions to a national sample of diverse youth regarding their perspectives on their access and willingness to pursue CT and NG screening and treatment.

## Methods

We used a longitudinal text message survey, MyVoice, of 1115 youth aged 14–24 years, to characterize youth perspectives on STI screening and treatment [5]. MyVoice participants are recruited to the survey cohort via targeted Facebook® and Instagram® advertisements based on demographic benchmarks (age, gender, race and ethnicity, and region of the country) from weighted samples of the American Community Survey [6]. Eligibility criteria include age 14–24 years, ability to understand and respond in English text, and access to a device with text messaging capabilities. MyVoice participants meet the minimum age requirement where parental consent is not required for STI services [7]. Once recruited, participants in the MyVoice cohort are asked open-ended questions via text message each week on a variety of health and health policy topics. Questions posed are iteratively developed by a team of physicians, methodologists, statisticians, and students to ensure clarity and ease of response for participants.

In August 2018, the MyVoice participants were prompted to respond to a set of four questions related to STI screening, treatment, and disclosure of their STI infections: 1) *We want to talk about chlamydia and gonorrhea, two of the most common sexually transmitted infections (STIs). Would it be hard for you to get TESTED for chlamydia or gonorrhea if you wanted?* 2) *If you tested positive, would it be hard for you to get TREATED for chlamydia or gonorrhea?* 3) *If you thought you had chlamydia or gonorrhea, where would you go to get treatment?* 4) *If you got chlamydia or gonorrhea, would you tell your sexual partner(s)? Why or why not?*

Grounded theory methodology was used to review the responses by identifying themes and iteratively developing a codebook that consisted of categories representing the most common responses. Two reviewers independently coded each question, with discrepancies resolved by a third reviewer. Summary statistics were calculated for demographic data collected at enrollment and included gender, race, U.S. geographic region [8], education level, and receipt of free or reduced school lunch (a proxy for socioeconomic status). The frequency of coded themes was also analyzed using summary statistics (Microsoft Corporation. 2016. *Microsoft Excel*). The mean age of individuals in each response category for question 1 and 2 were compared using t-test with *p* < 0.05 representing statistical significance (SAS Institute Inc. 2013. *SAS® 9.4)*.

## Results

Among the 800 participants who responded to at least one question (72% response rate), the mean age was 19 years (SD = 3.1), 55% identified as female, 61% identified as non-Hispanic white, and 33% qualified for free or reduced lunch (Table 1). Quotes from respondents are included below as representative examples of specific themes.

**Table 1:** Demographic characteristics of survey respondents and non-respondents from the MyVoice cohort

Characteristic	Respondents (***n*** = 800) n (%) or Mean (SD)	Non-Respondents (***n*** = 315) n (%) or Mean (SD)
Age	18.6 (3.1)	18.6 (3.1)
Gender		
Male	305 (38.1)	143 (45.8)
Female	443 (55.4)	144 (46.2)
Other gender	52 (6.5)	25 (8.0)
Race		
Asian	82 (10.3)	37 (11.9)
Black or African American	69 (8.6)	32 (10.3)
White or Caucasian	546 (68.3)	201 (64.4)
Mixed race	69 (8.6)	24 (7.7)
Other race	34 (4.3)	18 (5.8)
Ethnicity		
Hispanic or Latino	98 (12.3)	50 (16.0)
Non-Hispanic or Latino	702 (87.8)	262 (84.0)
Education Level		
Less than high school	434 (54.3)	158 (50.6)
High school graduate	70 (8.8)	35 (11.2)
Some college or tech school	185 (23.1)	70 (22.4)
Associate’s degree or tech graduate	21 (2.6)	9 (2.9)
Bachelor’s degree or higher	90 (11.3)	40 (12.8)
Region		
Midwest	389 (48.9)	172 (55.3)
Northeast	84 (10.5)	32 (10.3)
South	186 (23.3)	48 (15.4)
West	138 (17.3)	59 (19.0)
Free and reduced lunch eligibility		
Yes	263 (33.2)	91 (30.0)
No	530 (66.8)	212 (70.0)

### Most respondents felt it would be easy to get screened and treated for an STI

When prompted about ease of access to STI services, the majority of youth felt that it would not be difficult to both get screened (69%) and treated (68%) for an STI (Table 2). STI screening was reported to be easily accessible by the majority of respondents primarily because *“there are a lot of ways that you are able to get tested whether it be at the doctor or at a clinic”* and *“the doctor [is] close by and I believe testing is very cheap.”* A few respondents (*n* = 10) who believed they were not at risk or did not have STIs stated that *“It wouldn’t be hard for me to get tested because I know I do not have chlamydia or gonorrhea.”* For those who expressed difficulty in obtaining testing (21%), the most common reason was due to their minor status (37%) because they had *“been told by doctors in the past that they don’t know how STI tests show up on bills and my parents would be very upset to learn I was having sex.”* Respondents who reported that it was hard to get tested or treated for STIs (6.5%) were on average younger than those who reported no (i.e., no, unsure, and other) difficulty (17.4 years vs 19.0 years; *p* < 0.001 and 17.4 years vs. 18.9 years; p < 0.001, respectively). They commonly cited that *“I have no idea where I would go to get tested, so I’m not really sure if it would be difficult or not. I guess the first thing I would need to do is find out where or who would even have that kind of service”* (31%). See Additional file 1 for detailed response patterns.

**Table 2:** Questions, themes, and representative respondent quotes

Question, Theme	n (%)^**a**^	Representative Quote
**Would it be hard for you to get TESTED for chlamydia or gonorrhea if you wanted? (*n*** **= 782)**^**b**^		
No	539 (68.9)	
*Easy access to care*	385 (71.4)	“there are clinics everywhere”
*Access to insurance/funds*	81 (15.0)	“I have insurance so I could get tested free”
*Importance of health*	33 (6.1)	“No I want to see if I’m STD free”
Yes	162 (20.7)	
*Notification of parents*	60 (37.0)	“Yes, I am not going to tell my parents I’m sexually active”
*Embarrassment/stigma*	38 (23.5)	“There is some stigma”
*Unsure of process or location*	39 (24.1)	“Yes, as I am not sure how and where to get tested”
*Cost/insurance*	29 (17.9)	“I don’t have health insurance”
Unsure	51 (6.5)	
*Unsure of process or location*	16 (31.4)	“Probably not, but I don’t know where or how to”
**If you tested positive, would it be hard for you to get TREATED for chlamydia or gonorrhea? (*n*** **= 771)**^**b**^		
No	523 (67.8)	
*Access to insurance/funds*	216 (41.3)	“I have health insurance and am financially stable enough … “
*Easy access*	202 (38.6)	“I have access to many good medical facilities”
*Importance of health*	41 (7.8)	“that is the only option for me to get better and healthy”
Yes	122 (15.8)	
*Cost/insurance*	51 (41.8)	“My health insurance doesn’t cover STDs”
*Notification of parents*	30 (24.6)	“difficult to explain to parents”
*Embarrassment/stigma*	21 (17.2)	“The social stigma surrounding STIs would prevent me from asking for help about any treatment”
Unsure	122 (15.8)	
*Unsure of process*	29 (23.8)	“I don’t know enough about treatment.”
*Depends on cost/insurance*	23 (18.9)	“If my healthcare covers it, then no. If it doesn’t, that’s a different story.”
**If you thought you had chlamydia or gonorrhea, where would you go to get treatment? (*n*** **= 745)**^**b**^		
Doctor’s office	368 (49.4)	“… my PCP for a consultation and to get treated/tested”
Free clinic/Planned Parenthood	106 (14.2)	“Planned Parenthood or a walk-in-clinic if … at school”
Hospital	105 (14.1)	“university system or other local hospital”
Gynecology/STI doctor	85 (11.4)	“My gynecologist, preferably. If I couldn’t schedule an appointment for a while, I’d go to a clinic.”
Unsure	83 (11.1)	“I’m not sure, the doctor?”
School clinic	65 (8.7)	“If it was during the school year, I would start with on campus health services.”
**If you got chlamydia or gonorrhea would you tell your sexual partner(s)? Why or why not? (*n*** **= 774)**^**b**^		
Yes	736 (95.1)	
*Effects partner*	185 (23.9)	“it’s not something that only affects you, but anyone else you may have been with”
*Morality*	180 (23.3)	“Yes, that is the right thing to do”
*Importance of testing/treatment*	146 (18.9)	“Absolutely. He would need to get treated/tested too.”
No	16 (2.1)	
*Social stigma*	10 (62.5)	“I would be too ashamed”

^a^Numbers may not add to 100%, as codes are not mutually exclusive; not all codes are displayed

^b^N = the number of coded responses to each question; not all respondents answered each question

STI = sexually transmitted infection

Many participants (41%) noted that STI treatment would be easy to get because they *“have insurance and are financially stable enough to treat it.”* Respondents who thought treatment would be difficult to get (16%) had concerns about how *“my health insurance doesn’t cover STDs”* (42%) and *“I won’t feel so comfortable talking to my parents which I would probably have to I guess. But I wouldn’t know how to handle it myself”* (25%). Those who remained uncertain (16%) primarily indicated they *“don’t actually know what [their] options would be for treatment”* (24%).

### Primary care offices were the most common place youth would go for STI treatment

Youth indicated preference for STI treatment via primary care providers by stating that *“my doctor”* (49%), free clinics (14%), or hospitals (14%) were the main locations they would seek health services. Less common locations included a gynecologist (11%) and *“If it was during the school year, I would start with on-campus health services”* (9%). An additional 11% of respondents were unsure about where they would go for their STI treatment.

### Almost all respondents would share an STI diagnosis with their sexual partners

Nearly all respondents to this question (95%) also stated they would communicate their STI diagnosis with their sexual partners because *“they could have it too,” “it would be important for them to know and get tested and contact any other sexual partners to also be checked,”* and *“it’s the right thing to do*.*”* Those who would not disclose their status to their partner (2%) cited *“I would be too ashamed”* and *“it would be embarrassing”* as reasons for not informing their partner of their infection.

## Discussion

Our study found that most youth consider both STI screening and treatment to be accessible because of easy access to healthcare or access to insurance and funding. Additionally, youth in our sample noted a preference for going to their established doctors or primary care providers for treatment services. Most notably, we found that nearly all MyVoice youth respondents indicated they would confide in their partners about an STI diagnosis, with more than half of these respondents reporting reasons such as how the diagnosis impacts their partners and that it is morally right.

While other studies report that many youth have access to general healthcare, there is limited literature on youth knowledge of included health services [9, 10]. Our study provides insight here by noting that our cohort believes STI screening and treatment services to be accessible via established or local health providers. This highlights the importance of youth having a healthcare home at sites that they routinely encounter, such as school-based health centers and federally qualified health centers. During the COVID-19 pandemic, the use of telehealth services has increased to support wider access to health services, but virtual healthcare services may not replace necessary clinical services when inequities in technology access remain [11]. Harnessing the interest and willingness of youth to seek sexual healthcare services at locations they are comfortable with is critical given that access to reproductive health services continues to decline [12], despite the growing incidence of STIs among youth [13]. Similar to our findings, access to care and insurance has previously been noted to make screening and treatment easy for youth [14]. However, our data does not support limited knowledge of health services as the primary barrier to care. Youth in our sample noted other barriers in accessing these services like cost or insurance coverage, embarrassment, and concerns about notifying their parents.

Youth concerns about confidentiality regarding an STI diagnosis note difficulty “*… because it would be something that I would have to tell my parents and that would be very uncomfortable,”* or *“… because treatment would require health care, which would require me telling my parents.”* This is consistent with existing literature that reports how perceptions of confidentiality may pose a barrier to healthcare for youth [3, 4]. Addressing youth concerns about cost and confidentiality must be considered when developing and implementing STI treatment and prevention services. Providers and health departments can encourage positive communication about sexual health between parents and their children, thus promoting safer sex practices and better health outcomes [15, 16]. In addition, providers can educate youth on the rules of confidentiality between minor patients and providers.

Despite their perceived ease of access to STI screening and treatment, actual use of these services remains low among U.S. youth. A possible explanation for this discrepancy, as noted in previous work, may be due to youth assuming that they are STI-free or generally not at risk [4]. Our study supports this conclusion, with some respondents reporting *“It wouldn’t be hard for me to get tested because I know I do not have chlamydia or gonorrhea”* and *“No I go to the doctors often and they ask if I want to get tested but I’ve been with the same person since the last test I have no reason to get tested.”* Youth must also want to, or perceive a need to, get STI screening. This barrier to use of STI screening services illustrates the need for local and federal health officials to support initiatives that emphasize—to youth and providers—the importance of regular STI screening, even in asymptomatic individuals.

Findings from our work also suggest primary care offices as the preferred location for STI screening and treatment in youth. This is congruent with previous work on STI screening amongst youth in the U.S. that indicated the majority of those who sought STI screening were evaluated at primary care physicians’ offices [4]. Youth preferences for STI screening and treatment at primary care clinics and concern for costs may require additional support and education for primary care health professionals. Furthermore, STI screening and prevention counseling for youth during their routine clinic visits will serve to increase awareness of STI screening methods and treatment options.

Our study also reports the important finding that nearly all respondents (95%) stated they would share their STI results with their partners. Common responses included *“Yes because they need to know, in case they have it too. Also, they can help prevent the spread.”* and *“Yes it’s the most responsible thing to do. They would deserve to know.”* A previous study on sexual health behaviors of U.S. college-age men similarly reported that the majority of participants were willing to disclose their STI status to their partners [17]. Our results contrast with previous data on youth concerns of STI stigma and the general misperception of youth being less willing to notify their partners [3, 4, 18, 19]. Youth willingness to confide in their sexual partners and concern about health effects on their sexual partners supports potential use of expedited partner therapy (EPT) to increase treatment of STIs. EPT—a treatment option where individuals can obtain STI medications or prescriptions for their sexual partners—may provide a useful opportunity to support youth treatment as it is quick, convenient, and respects patient privacy [20]. Clinician education on youth willingness to share STI results and use of EPT may also be beneficial to their efforts to increase screening and treatment in their patient populations. Additionally, it may address the hesitancy and uncertainty that some clinicians may face regarding the permissibility of this therapeutic measure [21]. It is important to note that youth willingness to participate in partner notification does not necessarily reflect youth behaviors. Currently, there is a gap in literature on the rates of partner notification specifically in the youth population [22]. In an Australian evaluation of individuals > 16 years diagnosed with chlamydia (median age of 27 and 24 years in males and females, respectively), 31 and 46% of heterosexual males and females notified their partners [23]. However, partner notification is increased in youth (ages 13–20 years) with higher levels of self-efficacy and in relationships with stronger emotional ties [24]. This mirrors the findings noted in adult populations, where partner notification is highest for spousal partners than for causal or commercial partners [22]. Thus, further evaluations of partner notification in youth ages 15–24 years are needed to quantify the efficacy of interventions like EPT among youth.

**Fig. 1:**
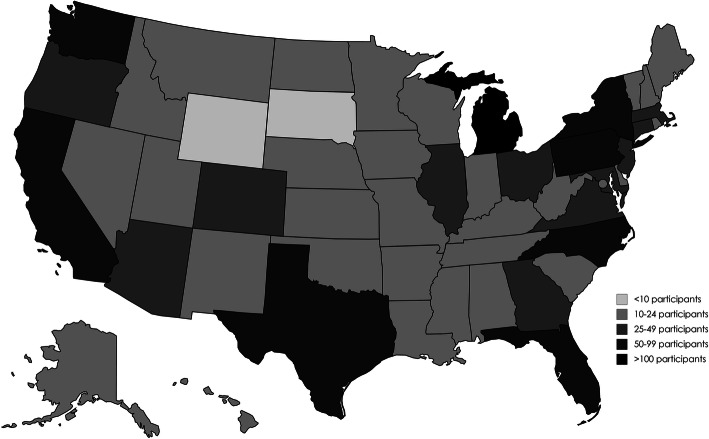
MyVoice survey participant heat map, by state

Though the MyVoice cohort sample recruits nationally from youth aged 14–24 years, there are some limitations. While MyVoice recruits based on benchmarks for national data on age, gender, race and ethnicity, and region of the country, respondents are not nationally representative because there is no assurance that the recruitment advertisements will reach all eligible participants. Additionally, recruitment via social media may bias the sample by including only those who use social media, limiting generalizability. Specifically, MyVoice respondents are oversampled in the Midwest region (Fig. 1) of the U.S., thereby providing data that may not necessarily reflect the perceptions and practices of youth in other parts of the country. Another limitation stems from the lack of concurrence between the time at which the survey was administered and analyzed and the onset of the COVID-19 pandemic. The majority of the cohort reported access to healthcare services at the time the survey was administered, yet youth have experienced decreased access to reproductive healthcare services during the COVID-19 pandemic [25]. To protect our respondents from having to self-report their past STI history, the open-ended questions posed to youth also asked about their theoretical behaviors. This may lead to desirability bias, as individuals may over- or under-report to conform to societal norms [26]. The other limitation of assessing theoretical behaviors is that intentions may not always lead to actions. Finally, the anonymous nature of this protocol prevented us from clarifying any unclear or missing responses. This is illustrated by our inability to discern if sexual partners were primary or casual in nature.

## Conclusions

Understanding youth insights on their sexual health perspectives is instrumental in mitigating the disproportionately high rates of STIs that affect this population. Our findings suggest that youth are committed to both their and their partners’ sexual health, presenting an invaluable opportunity for eventual large-scale intervention through partner-based referral and treatment options such as EPT. However, before such solutions can be explored, the discrepancy between youth intentions and actions must be addressed to provide insight on how to encourage positive behavioral change that could potentially reduce STI rates in this population. Longer-term societal efforts toward educating parents and youth on appropriate sexual health education, STI screening and treatment costs, and resources may support reduction in STI transmission [16, 27, 28]. Health and education departments can also partner to implement policies and programs that support and normalize regular STI screening. Ultimately, promotion of STI prevention services and reduction of the perceived barriers are needed to help combat the increasing STI incidence rates among youth.

## Supplementary Information

**Additional file 1.** Details of Q2 responses based on Q1 responses. Description: Details showing the relationship between the responses for those who completed question 2 in relation to their response to question 1.

## Data Availability

The datasets used and/or analyzed during the current study are available from the corresponding author on reasonable request.

## Abbreviations

STI: sexually transmitted infection
CDC: Centers for Disease Control and Prevention
CT: *Chlamydia trachomatis*
NG: *Neisseria gonorrhoeae*
