# Supplementary material for: The future of STI screening and treatment for youth: a National Survey of youth perspectives and intentions
Source: BMC Public Health. 2021 Nov 4;21:2006. doi: 10.1186/s12889-021-12091-y (PMC8567981; doi:10.1186/s12889-021-12091-y)
Supplement: Supplementary file 1 — Additional file 1. Details of Q2 responses based on Q1 responses. Description: Details showing the relationship between the responses for those who completed question 2 in relation to their response to question 1. [file 12889_2021_12091_MOESM1_ESM.docx]

**Additional file 1.** **Details of Q2 responses based on Q1 responses**

726 participants had coded responses to all four questions posed

People who responded the same way to both questions:

- Of the 539 participants who said it would not be difficult to get tested, 405 also reported it would not be difficult to get treated
- Of the 162 who said it would be difficult to get tested, 68 also reported it would be difficult to get treated
- Of the 51 who were unsure about testing, 18 were unsure about treatment

**Details of Q2 responses based on their categorized Q1 response***

|  | Not difficult treatment | Difficult treatment | Unsure treatment |
| --- | --- | --- | --- |
| Not difficult testing (n=539) | 405 (75.1%) | 38 (7.1%) | 79 (14.7%) |
| Difficult testing (n=162) | 74 (45.7%) | 68 (42.0%) | 18 (11.1%) |
| Unsure testing (n=51) | 24 (47.1%) | 6 (11.8%) | 18 (35.3%) |

*Responses do not total 100 and do not match the Q2 totals, as these are just looking at the individuals who had a Q1 response followed by a Q2 response that was coded in one of the three categories.
